# Same-day Smear Method Compared with Conventional Sputum Method for Diagnosing Pulmonary Tuberculosis

**DOI:** 10.7759/cureus.2761

**Published:** 2018-06-07

**Authors:** Adimulam Ganga Ravindra, Manju Rajaram, Dharm Prakash Dwivedi, Madhusmita Mohanty Mohapatra, Chinnakali Palanivel, Jayalakshmi Ramakrishnan, Vinod Kumar Saka

**Affiliations:** 1 Jawaharlal Institute of Postgraduate Medical Education and Research (JIPMER), Puducherry, IND; 2 Pulmonary Medicine, Jawaharlal Institute of Postgraduate Medical Education and Research (JIPMER), Puducherry, IND; 3 Preventive Medicine, Jawaharlal Institute of Postgraduate Medical Education and Research (JIPMER), Puducherry, India.

**Keywords:** early morning, spot sample, tuberculosis, microscopy, diagnosis, sputum sample

## Abstract

Background

Patient compliance with the two-day Revised National Tuberculosis Control Programme’s (RNTCP) diagnostic process for pulmonary tuberculosis (TB) is poor in high case load settings, with a high dropout rate observed on the second day. Hence, the World Health Organization (WHO) has recommended the same-day (spot-spot) sputum test for high-burden TB countries to help reduce diagnostic dropouts. This study addresses the paucity of comparative data on the accuracy and agreement of the two methods, while the WHO recommendations are yet to be implemented by the RNTCP. The objective of this study was to assess and compare the smear positivity rates of the same-day and conventional sputum examination methods for the diagnosis of sputum smear-positive pulmonary TB.

Methodology

We conducted a cross-sectional, analytical, nonrandomized comparative study on presumptive TB patients attending a designated microscopy center in a tertiary care hospital. Three sputum samples were collected: a first spot, a second spot (one hour after the first spot), and an early morning sample taken on the following day. The first and the second spot samples taken one hour apart were included for microscopic analysis. The conventional (i.e., two-day sputum) method used the first spot and the early morning sputum sample taken on the following day. A positive result from any one of the three sputum samples was recorded as a proven TB case. We then compared the results of the smear microscopy obtained by the two methods.

Results

The same-day sputum microscopic method diagnosed 181 out of a total 189 TB cases. The conventional method diagnosed 188 cases. Thus, same-day sputum microscopy missed eight cases, whereas the conventional method missed only one case. The sputum positivity rate was 18.8% in the same-day sputum microscopy samples and 19.5% in the conventional method samples. The incremental yield of the second sputum sample in the same-day (second spot) sample was five cases (2.7%). In the conventional method (early morning sample), the yield was 12 cases (6.3%). The sensitivity of the same-day microscopy and conventional methods were 95.76% and 99.5%, respectively.

Conclusion

The conventional method of diagnosing sputum-positive pulmonary TB had more sensitivity compared to the same-day sputum microscopy approach.

## Introduction

Over six million new cases of tuberculosis (TB) are diagnosed each year around the world, and each year witnesses 1.3 million TB-related deaths [1]. A patient having active pulmonary TB expels infectious droplets measuring 0.5–5 microns while coughing, sneezing, and speaking [2]; even a single bacterium can cause a new infection. One smear-positive patient can infect 10–15 people in a year, and 10% of them will develop this disease [2]. Normally, the transmission can be stopped by early diagnosis and treatment with effective anti-TB agents.

The passive case finding method used in the Revised National Tuberculosis Control Programme (RNTCP) requires presumptive TB patients to visit healthcare facilities multiple times to give spot sputum samples on the first day, give early morning samples on the second day, and collect the report on subsequent days. In the conventional sputum smear microscopy method (spot-morning), patient compliance is poor; up to 50% of the patients fail to return to either provide a second specimen or receive the results. The increased dropout rate may be due to the significant amount of money needed for accommodation, food, and travel to the designated microscopy center in addition to the lost daily wages for time away from work. These patients are known as “diagnostic defaults” [2].

In 2011, the World Health Organization (WHO) recommended that the countries already implementing the two-specimen case finding (by spot and early morning) should switch to the same-day (spot-spot) diagnosis, especially in settings where patients are likely to default from the diagnostic pathway [3]. Not requiring patients to return the following day can significantly reduce the economic burden of the patient, thereby resulting in lower dropout rates during diagnosis. In India and other countries, only a few studies have addressed the accuracy and agreement of the positivity rates of the same-day sputum smear microscopy when compared to the conventional method, and the results of those few studies are conflicting. Same-day sputum smear microscopy may have advantages over conventional sputum smear microscopy in increasing the TB case detection and reducing the number of diagnostic defaults [4]. Therefore, we compared the smear positivity rate and the agreement between the same-day sputum smear (spot-spot) microscopy and the conventional sputum smear (spot-morning) microscopy in the diagnosis of pulmonary TB.

## Materials and methods

This was a cross-sectional comparative study among patients attending the outpatient pulmonary department (OPD) of a tertiary care postgraduate research institute from June 2015 to December 2016. All patients attending the OPD were screened for presumptive TB, and they were included in the study for validity assessment.

**Figure 1:**
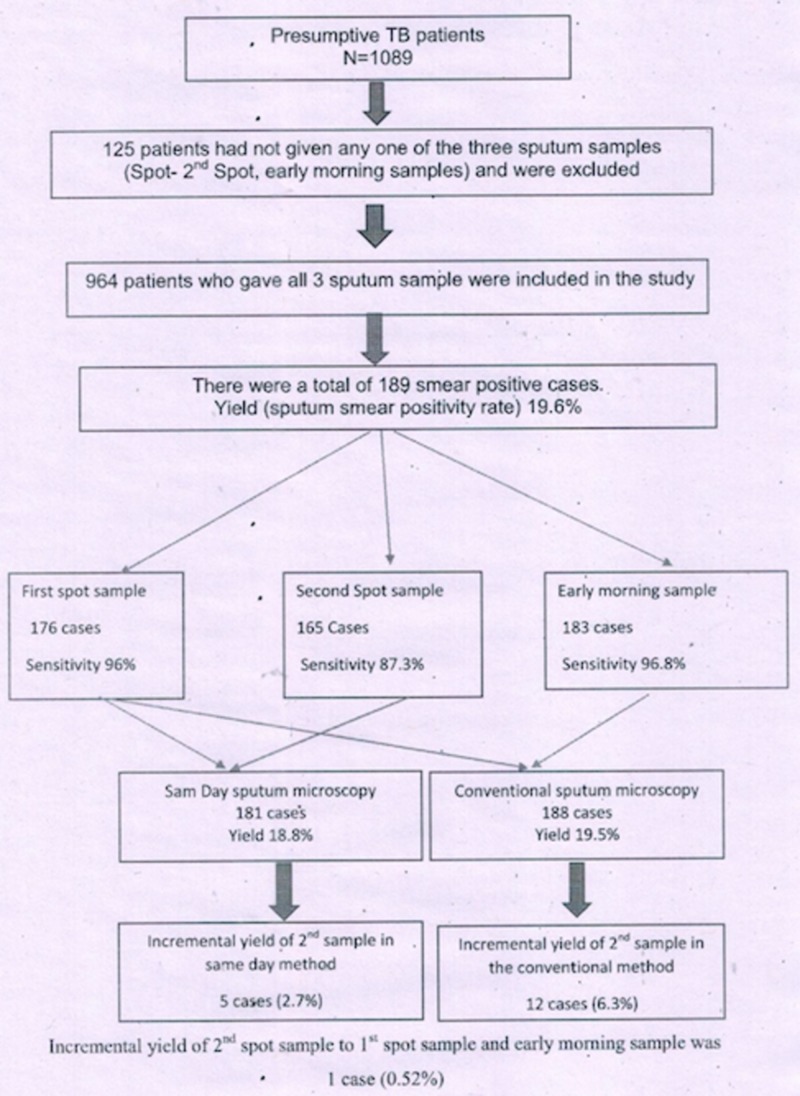
Flow chart of presumptive tuberculosis (TB) patients diagnosed to be confirmed TB cases by same-day sputum microscopy and conventional sputum microscopy.

After providing informed written consent, patients were evaluated according to the flowchart presented in Figure 1. We collected demographic information from eligible patients using pre-structured proforma. Patients were informed about the study, and they were asked to give three sputum specimens: first spot, second spot (after one hour), and next day early morning specimens. Patients were asked to take a deep breath and cough properly to bring out good quality sputum. Sputum were stained by auramine-phenol staining and examined by a single laboratory technician at the Jawaharlal Institute of Postgraduate Medical Education and Research (JIPMER), our designated microscopy center. The results of the first spot and the second spot samples comprised the same-day approach samples, while the results of the first spot and the early morning samples comprised the two-day approach samples. The total number of cases diagnosed by the same-day (spot-spot) specimens and the number of cases diagnosed by the conventional (spot-early morning) specimens were computed, and the results were analyzed (Figure 1).

Sputum smear results were expressed as frequency and percentages. The comparison of the smear positivity results between the two methods was made by the exact McNemar's test. The validity of the spot sample was assessed by estimating the sensitivity and specificity along with the predictive values by treating the conventional method as the standard method. The incremental yield of the second specimen (i.e., instances when the result of the first specimen was negative and the second specimen was positive) was expressed as a percentage of all smear-positives diagnosed. All statistical methods were carried out at a 5% level of significance (p < 0.05).

## Results

There were 1089 patients suspected to have TB, among which 964 (88.5%) gave all three sputum samples. Among 964, 189 (19.6%) patients were found to be sputum-positive TB cases. Among those who gave all three samples, 46.6% were aged under 45 years old, and 62.6% were male patients. Among those sputum-positive cases, 49.7% were aged under 45 years old, and 79.4% were male patients.

The sputum positivity rate in the same-day group was compared with the conventional smear microscopy group, and the positivity rate did not have a significant difference (Table 1). The difference between the two methods in identifying TB cases was statistically significant (p = 0.0391, exact McNemar’s test).

**Table 1:** Sputum positivity rate (yield) in same-day (spot-spot) sputum smear microscopy versus conventional (spot-early morning) sputum smear microscopy (n = 964 presumptive tuberculosis patients).

Method	Number of cases diagnosed	Sputum positivity rate (yield) (%)	Exact McNemar’s test
Same-day sputum smear microscopy	181	18.8	p = 0.0391
Conventional sputum smear microscopy	188	19.5	
Total number of cases diagnosed	189	19.6	

The same-day sputum smear microscopy (spot-spot sputum sample) had a sensitivity of 95.7%, a specificity of 99.9%, and a positive predictive value of 99.44% when compared with conventional sputum smear microscopy (Table 2).

**Table 2:** Comparison of same-day sputum smear microscopy versus conventional sputum smear microscopy.

Same-day sputum smear microscopy	Conventional sputum smear microscopy		Total
	Positive	Negative	
Positive	180	1	181
Negative	8	775	783
Total	188	776	964

The same-day sputum smear microscopy (spot-spot sputum sample) had a sensitivity of 95.8%, a specificity of 100%, and a positive predictive value of 99.44% when compared with total TB cases (Table 3).

**Table 3:** Sensitivity and specificity of same-day sputum smear microscopy and conventional sputum smear microscopy.

	Same-day versus conventional microscopy (%)	Same-day versus total tuberculosis cases (%)
Sensitivity	95.7	95.8
Specificity	99.9	100
Positive predictive value	99.44	99.4

**Figure 2:**
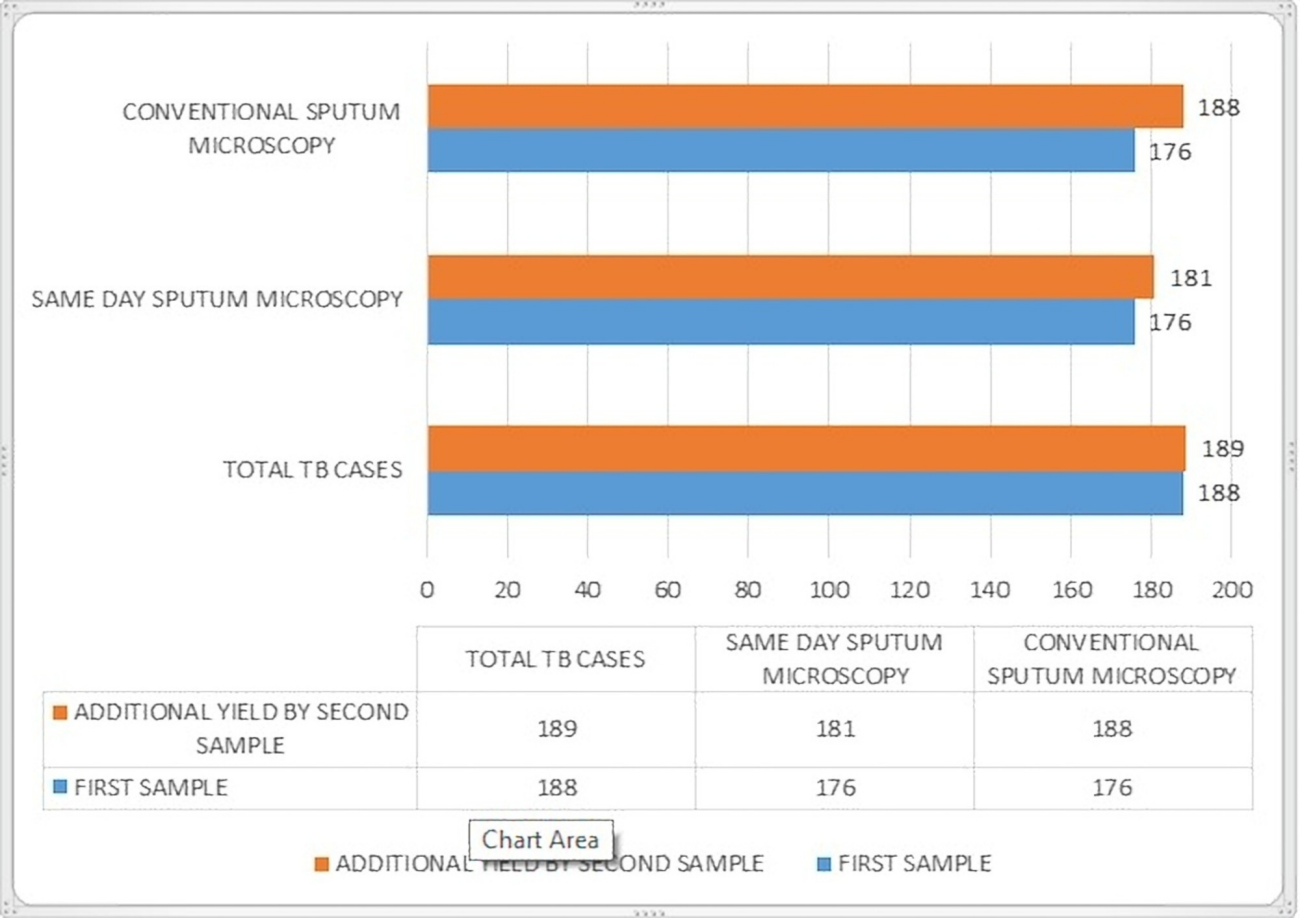
Incremental yield of the second sample to the first sample (n = 189).

The incremental yield was five cases (2.7%) for the second sputum sample in the same-day sputum microscopy and 12 cases (6.3%) for the conventional sputum smear microscopy. The additional yield of the second spot sputum sample compared to the first spot sputum sample and early morning sputum sample was one case (0.52%) (Figure 2).

## Discussion

The sputum positivity rate of this study was 19.6%, which aligns with similar positivity rates reported for other TB-prevalent countries (e.g., Nigeria, 21%; Ethiopia, 21%; Nepal, 25%; and Yemen, 24%) [5].

In TB control programs, the goal is to identify all sputum-positive cases, which are the potential sources of transmission of infection. Because the early morning sputum sample has the highest sensitivity, and it is of better quality compared to the spot samples, we can infer that the early morning sample is the preferred sample compared to the spot samples. Cuevas et al. arrived at a similar inference; they reported that the early morning sputum sample is more sensitive because of its high bacillary load [6-8]. The yield of the same-day microscopy was 18.8% (181 of 964), and the yield of the conventional microscopy was 19.5% (188 of 964). We found that while the same-day sputum smear microscopy missed eight cases (4.2%), conventional sputum smear microscopy missed only one case (0.5%). Myneedu et al. found that 43 out of 330 presumptive TB patients (13.03%) were smear-positive according to the same-day method, while they found the conventional method yielded 61 (18.48%) smear-positive cases [9]. In the same-day sputum examination, 6% of lesser bacilli were identified compared to the conventional method. They confirmed these findings by culture, which showed that the same-day sputum approach was less sensitive than the conventional method [9].

Nayak et al. conducted a study across seven district level hospitals in Chhattisgarh, India, and found that 433 out of 2551 presumptive TB patients were sputum-positive. Some 360 (14%) patients were found smear-positive by the same-day microscopy, and 431 (17%) patients were found smear-positive by the conventional method (p < 0.001). A total of 73 (16.9%) potential cases were missed by the same-day method compared to only two cases (0.5%) missed by the conventional method [6].

All these studies found that the sensitivity of same-day microscopy was statistically significantly lower compared to conventional microscopy. The proportion of cases missed by the same-day microscopy was relatively higher (16.9%) in the study by Nayak et al. compared to both the study by Deka et al. (6.5%) and this study (4.2%). However, the proportion of cases missed by conventional microscopy in all these studies was the same (0.5%), which is negligible [6-7].

In all three studies (including this study), the incremental yield of the second specimen is higher in the conventional method (i.e., the early morning specimen) compared to the same-day method (second spot specimen). In this study, going by the absolute numbers, the conventional method could pick up seven more sputum-positive cases compared to the same-day method, which bears clinical significance. Only one additional case (0.5%) could be diagnosed by the second spot sample missed by the first spot and the early morning sample. The second spot sample offers no diagnostic advantage compared to the first sample and the early morning sample. However, as there is an incremental yield of five cases in this study by the second spot sample in the same-day approach, patients who may not be able to come for the following day’s test should be encouraged to give the second spot sample.

These studies used an adequate sample size; however, failure to blind the samples for the laboratory technician could have introduced bias. Considering all these facts, we can reasonably conclude that the conventional approach, which involves collecting two sputum samples (spot-early morning) is far more sensitive, and it is much more useful diagnostically, compared to the same-day (spot-spot) approach.

Our conclusion is in stark contrast to the WHO policy statement and the results from other studies [3, 5, 10-15] concluded that the same-day microscopy has a diagnostic accuracy similar to the conventional microscopy.

The WHO policy statement was based on a meta-analysis by Davis et al., which did not include studies from India. Hence, the WHO recommendation may not apply to India. When analyzing other studies from India and other countries, it is evident that all the studies used a very small sample size ranging from 200 to 400 presumptive TB cases, and most of the results were not statistically significant. This might be the reason for the apparent equal efficacy of the same-day and the conventional method presented in those studies [3, 5, 10-15].

The disadvantage of the conventional approach has always been the multiple visits required from the patient’s side for the early morning sample to be taken on the following day. However, considering the increased diagnostic yield from the early morning sample, the patients should be properly educated and counseled regarding the importance of returning to provide the early morning sample. Given that the patients’ arrive from a radius of 100 km to a tertiary care center like JIPMER, and 91.8% (989 out of 1089) return the next day to give the early morning sample, a similar low rate of patient defaults/high rate of patient return can be achieved in primary health centers.

## Conclusions

The conventional approach of collecting two sputum samples (spot-early morning) is more sensitive, and it is much more useful diagnostically compared to the same-day (spot-spot) approach for diagnosing sputum-positive pulmonary TB patients.

## References

[1] World Health Organization (2018). Global Tuberculosis Report - 2017. http://www.who.int/tb/publications/global_report/Exec_Summary_13Nov2017.pdf?ua=1.

[2] Juyal D, Thaledi S (2014). Same day sputum microscopy: optimizing the diagnosis of pulmonary tuberculosis. OA Case Rep.

[3] World Health Organization (2011). Same-Day Diagnosis of Tuberculosis by Microscopy: WHO Policy Statement. http://www.stoptb.org/wg/gli/assets/documents/WHO%20Policy%20Statement%20on%20Same-day-diagnosis%20by%20microscopy%20FINAL%20March%202010.pdf.

[4] Khan MS, Khan S, Godfrey-Faussett P (2009). Default during TB diagnosis: quantifying the problem. Trop Med Int Health.

[5] Ramsay A, Yassin MA, Cambanis A (2009). Front-loading sputum microscopy services: an opportunity to optimise smear-based case detection of tuberculosis in high prevalence countries. J Trop Med.

[6] Nayak P, Kumar AMV, Claassens M (2013). Comparing same day sputum microscopy with conventional sputum microscopy for the diagnosis of tuberculosis -- Chhattisgarh, India. PLoS One.

[7] Deka DJ, Choudhury B, Talukdar P (2016). What a difference a day makes: same-day vs. 2-day sputum smear microscopy for diagnosing tuberculosis. Public Health Action.

[8] Cuevas LE, Yassin MA, Al-Sonboli N (2011). A multi-country non-inferiority cluster randomized trial of frontloaded smear microscopy for the diagnosis of pulmonary tuberculosis. PLoS Med.

[9] Myneedu VP, Verma AK, Sharma PP, Behera D (2011). A pilot study of same day sputum smear examination, its feasibility and usefulness in diagnosis of pulmonary TB. Indian J Tuberc.

[10] Hirao S, Yassin MA, Khamofu HG (2007). Same-day smears in the diagnosis of tuberculosis. Trop Med Int Health.

[11] Shafiyabi S, Ravikumar R, Ramaprasad Ramaprasad, Krishna S (2013). Study of same day sputum smear examination in diagnosis of pulmonary tuberculosis under RNTCP. Sch Acad J Biosci.

[12] Chandra TJ (2012). Same day sputum smear microscopy approach for the diagnosis of pulmonary tuberculosis in a microscopy centre at Rajahmundry. http://www.tbassnindia.org/forms/Binder2.pdf#page=18.

[13] Davis JL, Cattamanchi A, Cuevas LE, Hopewell PC, Steingart KR (2013). Diagnostic accuracy of same-day microscopy versus standard microscopy for pulmonary tuberculosis: a systematic review and meta-analysis. Lancet Infect Dis.

[14] Chandra TJ, Raj RS, Sharma YV Same day sputum smear microscopy approach with modified ZN staining for the diagnosis of pulmonary tuberculosis in a microscopy centre at Rajahmundry. Indian J Med Microbiol.

[15] Islam MR, Khatun R, Uddin MKM (2013). Yield of two consecutive sputum specimens for the effective diagnosis of pulmonary tuberculosis. PLoS ONE.

